# Recent advances in nano-drug delivery systems for synergistic antitumor immunotherapy

**DOI:** 10.3389/fbioe.2022.1010724

**Published:** 2022-09-08

**Authors:** Bonan Zhao, Xiang Li, Ying Kong, Wenbo Wang, Tingting Wen, Yanru Zhang, Zhiyong Deng, Yafang Chen, Xian Zheng

**Affiliations:** ^1^ Leiden Academic Centre for Drug Research (LACDR), Leiden University, Leiden, Netherlands; ^2^ Department of Central Laboratory and Precision Medicine Center, Department of Nephrology, The Affiliated Huai’an Hospital of Xuzhou Medical University and Huai’an Second People’s Hospital, Huai’an, China; ^3^ Department of Radiology, Affiliated Hospital of Xuzhou Medical University, Xuzhou, China; ^4^ Department of Pharmacy, Affiliated Kunshan Hospital of Jiangsu University, Kunshan, China; ^5^ Department of Pathology, Affiliated Kunshan Hospital of Jiangsu University, Kunshan, China

**Keywords:** cancer therapy, immunotherapy, synergistic treatment, nano-drug delivery systems, nanomedicine

## Abstract

Immunotherapy has demonstrated great clinical success in the field of oncology in comparison with conventional cancer therapy. However, cancer immunotherapy still encounters major challenges that limit its efficacy against different types of cancers and the patients show minimal immune response to the immunotherapy. To overcome these limitations, combinatorial approaches with other therapeutics have been applied in the clinic. Simultaneously, nano-drug delivery system has played an important role in increasing the antitumor efficacy of various treatments and has been increasingly utilized for synergistic immunotherapy to further enhance the immunogenicity of the tumors. Specifically, they can promote the infiltration of immune cells within the tumors and create an environment that is more sensitive to immunotherapy, particularly in solid tumors, by accelerating tumor accumulation and permeability. Herein, this progress report provides a brief overview of the development of nano-drug delivery systems, classification of combinatory cancer immunotherapy and recent progress in tumor immune synergistic therapy in the application of nano-drug delivery systems.

## 1 Introduction

Tumors have surpassed cardiovascular diseases and become the leading cause of death worldwide, threatening human health and life (Mattiuzzi and Lippi, 2019; Bray et al., 2021). According to statistics from the World Health Organization, there were 18.1 million new cancer cases and 9.6 million cancer-related deaths globally in 2018 (Yates et al., 2020). In recent years, advances in immunotherapy have resulted in great improvements in cancer treatment (Yang, 2015). It is well known that the immune system plays an important role in cancer therapy, and several immunotherapies such as immune checkpoint inhibitors (Bagchi et al., 2021), adoptive immunotherapy (Lee, 2019) and tumor vaccines (Rammensee et al., 2020) have emerged as effective therapeutic strategies for treating cancer patients. However, tumor immunotherapy still encounters serious challenges, wherein certain tumors barely respond to immunotherapy. The lack of immunogenicity and subsequent insufficient antitumor immune response is a major reason for the lack of efficacy of several immunotherapies (Pilla et al., 2018). Therefore, it is important to seek strategies to strengthen the immunogenicity of tumors to enhance the efficacy of immunotherapy and overcome immune tolerance and escape.

**FIGURE 1:**
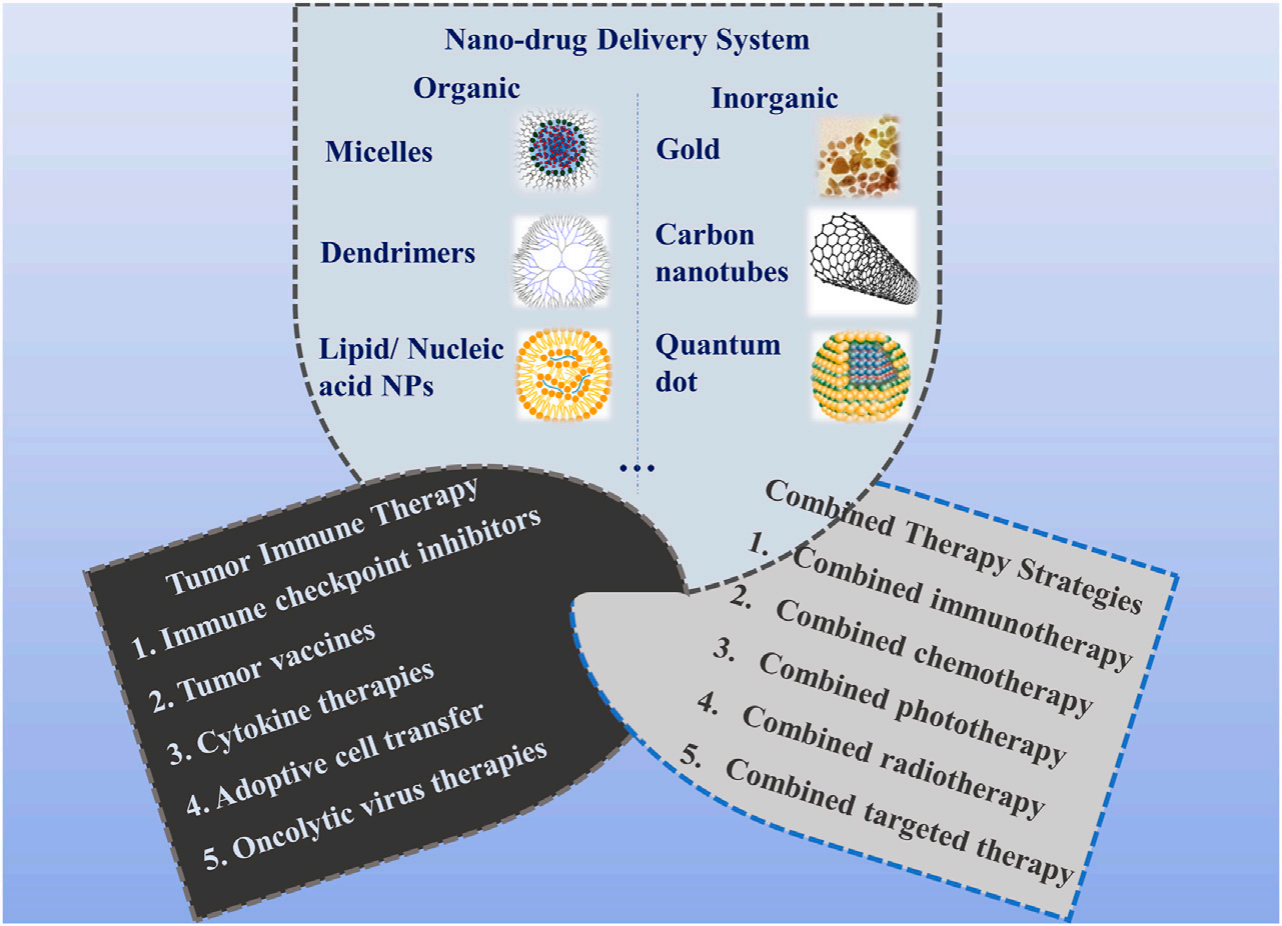
Classification of nano-drug delivery systems for synergistic antitumor immunotherapy.

In recent years, increasing attention has been given to combination therapies (Bayat Mokhtari et al., 2017), especially in the nanomedicine area (Jin et al., 2020a). Compared to immunotherapy alone, synergistic treatments, such as in combination with chemotherapy or phototherapy, offer multiple advantages with fewer side effects by avoiding multidrug resistance. Thus, it is necessary to combine immunotherapies with other therapeutic strategies. Among them, nanomedicine is one of the most important therapeutic approaches, which has been extensively applied in the clinic and pre-clinical investigations (Bayda et al., 2019). Nanoparticles (NPs) are the key components of nanomedicine and have received wide interest as promising drug-delivery systems for cancer treatment. Nanoparticles applied as drug delivery systems refer to nanoscale (usually 10–200 nm in diameter) particles, devices, or systems synthesized from various materials (Jin and Ye, 2007), including polymers (micelles, nanobrushes), lipids (liposomes), etc. (Bauer et al., 2021; Kappel et al., 2021; Zhang et al., 2022). They can increase the intracellular concentration of drugs within the cancer cells while decreasing their toxicity to the normal cells through passive or active targeting strategies, which are greatly helpful for increasing tumor immunogenicity by inducing T cell-mediated immune responses, thereby achieving high antitumor efficacy (Huang et al., 2021a). Considerable efforts have been made to develop novel nanoparticle-assisted cancer therapies in recent years. In this review, we summarize the recent advance in the development of nano-drug delivery systems for synergistic antitumor immunotherapy [Figure 1].

## 2 Overview of nano-drug delivery systems for cancer therapy

In recent years, nano-drug delivery systems have rapidly developed with the application of nanotechnology in medicine, which can deliver therapeutic agents to the target site, including proteins, nucleic acids (Torres-Vanegas et al., 2021), small molecule chemotherapeutics (Wang et al., 2020), and imaging agents (Ehlerding et al., 2018). The therapeutic agent can be integrated into the nano-drug carrier through covalent bonding (Rianasari et al., 2013), physical packaging (Huang et al., 2021b), electrostatic force (Zhang et al., 2018a) or coordination complexation (Siemer et al., 2021), thereby solving the limitations of conventional chemotherapeutic drugs, such as low water solubility (Merisko-Liversidge and Liversidge, 2008), instability in physiological conditions (Muthu and Feng, 2009), drug resistance (Siemer et al., 2021) and high toxicity (De Jong and Borm, 2008). Moreover, size effect of nanomedicine influences the pharmacokinetics of the drug, cellular uptake and penetration and accumulation in tumor tissues (Barua and Mitragotri, 2014). Besides, it can deliver tracer drugs and therapeutic drugs at the same time to realize the integration of tumor diagnosis and treatment (Jin et al., 2020b). Last but not least, co-delivery of multiple drugs or combined treatment with the second drug confers a synergistic antitumor therapeutic effect (Ma et al., 2013).

When it comes to the approaches to drug delivery *in vivo*, there are usually two ways that include passive and active targeting effects (Attia et al., 2019). On one hand, it is reported that size of NPs which range from approximately 40–400 nm is suitable to ensure long circulation time, enhanced accumulation in tumors with reduced renal clearance and is also known as enhanced permeation and retention (EPR) effect. Therefore, passive targeting is dependent on physiological features of tumor microenvironment (TME) like the abnormal vasculature, the surface charge of tumor cells, pH value and temperature (Shi et al., 2020; Wu, 2021). Although passive targeting has been widely developed and used, it is still faced numerous limitations such as the random targeting effects, which may result in insufficient drug diffusion into tumors. On the other hand, compared to passive targeting effects, active targeting effect could significantly increase the quality of delivery effects to target tumor cells. It can be achieved through the decoration of nano-drug carrier surfaces with ligands binding to receptors overexpressed onto tumor cells. Among the targeting ligands, folate, transferrin and epidermal growth factor receptors (EGFRs) have been widely used for the development of active targeting effect **(** Bertrand et al., 2014; Bazak et al., 2015).

### 2.1 Classification of nano-drug delivery systems

**FIGURE 2:**
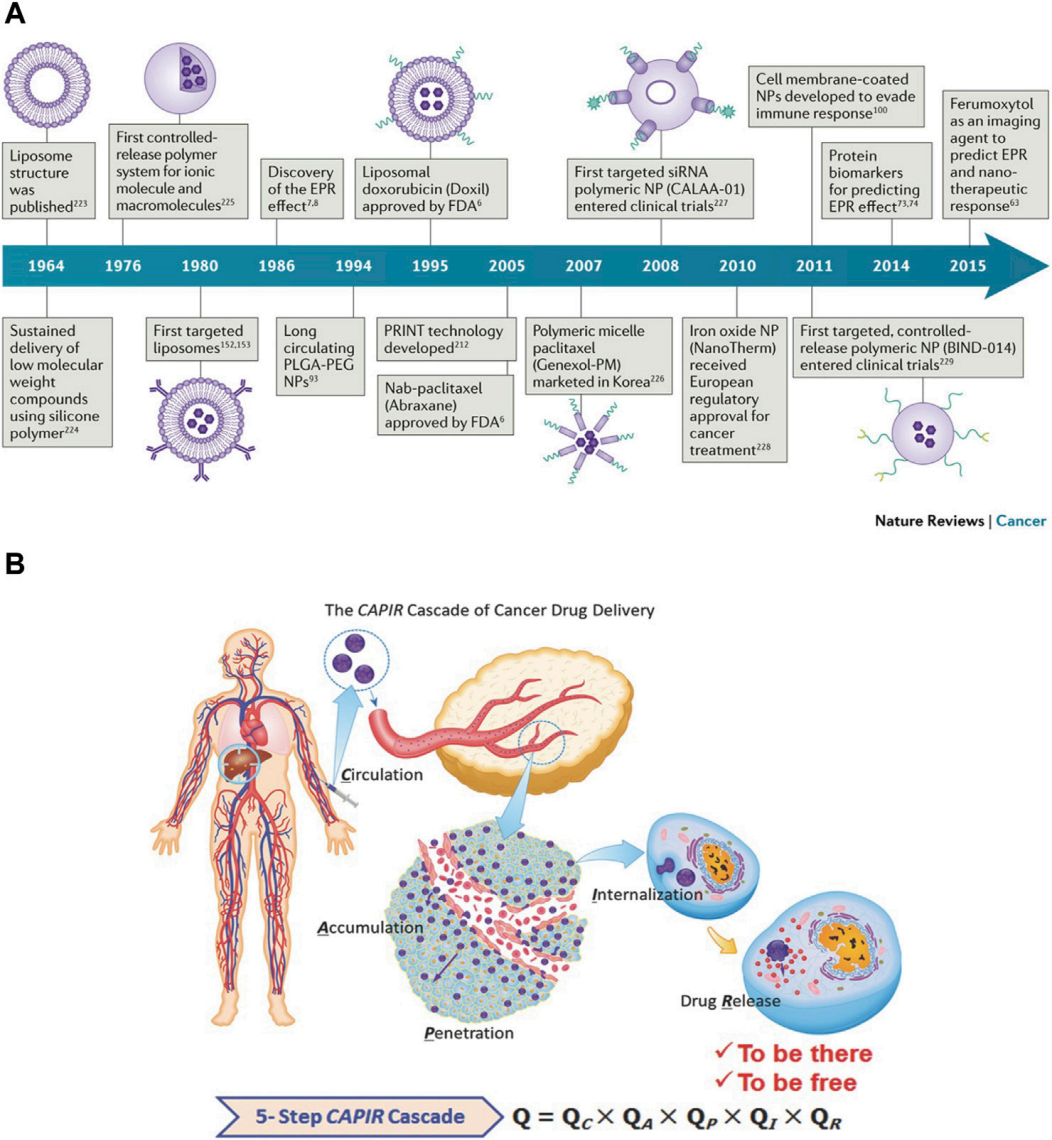
**(A)** Historical timeline of major developments in nano-drug delivery systems for cancer therapy (Shi et al., 2017). **(B)** A sketch of the CAPIR cascade of a nanomedicine to deliver a free drug into cancer cells (Sun et al., 2017).

In the past few decades, nanotechnology has made major contributions to the oncology field. Nano-drug delivery systems have progressed several generations from liposomes to the discovery of EPR effect, nucleic acid nano-medicines (siRNA), targeted controlled release polymer nanoparticles, cell membrane coated nanoparticles, and nanoimaging agents, etc [Figure 2A, (Shi et al., 2017),]. According to the source of the materials, they can be divided into natural carriers and synthetic carriers. With regards to their composition, they can be divided into organic nano-carriers, inorganic nano-carriers and composite nano-carriers. Organic nanoparticles are made of organic materials, especially carriers based on lipids, viral capsids, polysaccharides or protein particles (Virlan et al., 2016). Inorganic nano-carriers include metal nanoparticles like gold or silver, ceramic nanoparticles, quantum dots (fluorescent semiconductor particles), and carbon particles (single-wall or double-wall carbon nanotubes) (Auffan et al., 2009). With the development of more novel nanocarriers, it may be possible further expand the clinical and translational applications of nanomedicine.

### 2.2 Systemic delivery of nano-medicine

After systemic administration, nanoparticles need to overcome multiple obstacles before entering the tumor cells to exert their therapeutic effects. At first, the nanoparticles in the blood circulation easily interact with plasma proteins and are taken up by the reticuloendothelial system (RES) (such as liver and spleen) (Nie, 2010). Therefore, the nanoparticles in circulation must first escape the RES and then should be enriched in the tumor tissue. Afterward, when the nanoparticles reach the tumor site, they also have to pass through two obstacles. Firstly, they need to be transported across the tumor blood vessels. Although leaky and tortuous tumor blood vessels allow nano-medicine enrichment, parietal cells such as pericytes and basement membranes limit the exudation of nano-medicine through openings in the capillary wall, thereby reducing the convective transport of nano-medicine. Secondly, the dense extracellular matrix (ECM), owing to its high osmotic pressure, inhibits the passive diffusion of nano-medicine. To conclude, the entire delivery process of nano-medicine after systemic administration can be summarized by “CAPIR”: circulation, accumulation, penetration, internalization and release. Each step is independent and interconnected, involves a complicated process, and may affect the nanomedicine’s ultimate antitumor efficacy [Figure 2B, (Sun et al., 2017)].

## 3 Synergistic anticancer immunotherapy

### 3.1 Classification of tumor immunotherapy

**FIGURE 3:**
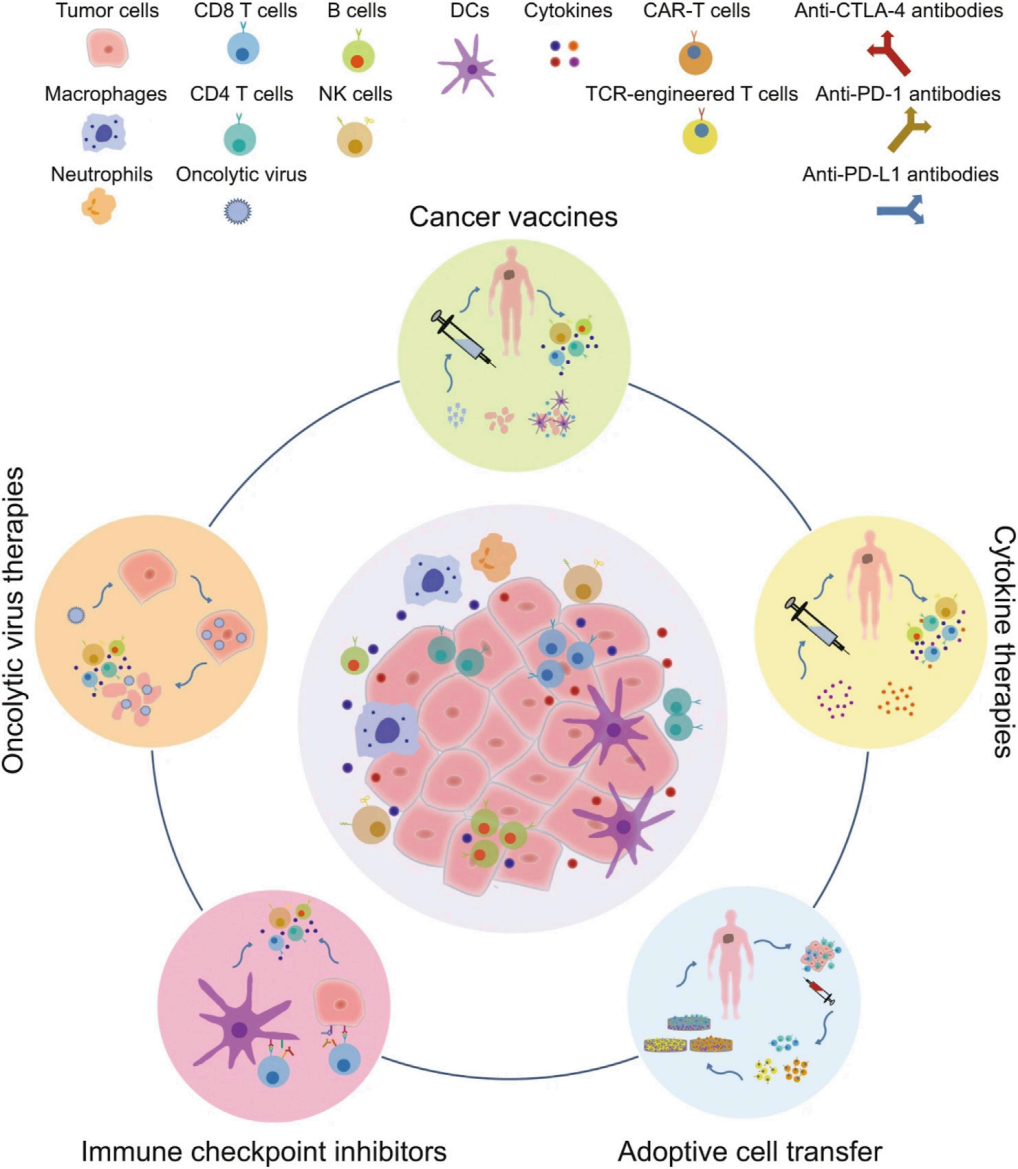
The major clinical categories of immunotherapy include oncolytic virus therapies, cancer vaccines, cytokine therapies, adoptive cell transfer, and immune checkpoint inhibitors (Zhang and Zhang, 2020).

Tumor immunotherapy is a new type of treatment, which is different from conventional radiotherapy and chemotherapy. Different from killing tumor cells directly, it aims to activate the body’s immune system and relies on the host’s immune function to kill the tumor cells (Zolnik et al., 2010), which has strong specificity, remarkable curative effects and long-term effects. Therefore, it has received great attention in the field of cancer therapy. Based on clinical cancer immunotherapy strategies, it can be divided into five categories, including immune checkpoint inhibitors, tumor vaccines, cytokine therapies, adoptive cell transfer, and oncolytic virus therapies [Figure 3, (Zhang and Zhang, 2020)].

#### 3.1.1 Immune checkpoint inhibitors

Immune checkpoint is a kind of immunosuppressive molecule, which plays a vital role in the maintenance of auto-immune tolerance and prevents normal tissues from being attacked by the immune system (Korman et al., 2022). However, during the development of cancers, tumor cells induce the high expression of immune checkpoint receptors through a variety of mechanisms to inhibit the function of T cells, thereby inhibiting the cytotoxic effect of the immune system and achieving tumor immune escape (Beatty and Gladney, 2015). Currently, the most widely used immune checkpoint inhibitors target the programmed death receptor-1 (PD-1), programmed death ligand-1 (PD-L1), cytotoxic T lymphocyte-associated antigen-4 (CTLA-4), lymphocyte activation gene-3 (LAG-3), which reactivate the tumoricidal effects of the immune system by interacting with the respective immune checkpoints to achieve antitumor immunotherapy (Franzin et al., 2020).

#### 3.1.2 Tumor vaccines

As early as the end of the 19th century, William B. Coley, the father of tumor immunology, used the toxin secreted by Streptococcus to treat cancer and opened the door for tumor vaccines for the first time (McCarthy, 2006). Tumor vaccines use tumor antigens to enhance the ability of antigen-presenting cells (APCs), activate antigen-specific effects, and the tumoricidal effects of T cells. There is no specific way to classify tumor vaccines. According to the specific uses of the tumor vaccines, they can be divided into two types: preventive vaccines (HBV, HPV) and personalized therapeutic vaccines (mRNA vaccine, DC vaccine) (Hollingsworth and Jansen, 2019). After the administration of the tumor vaccines, APCs present them to MHC II or MHC I and antigen-loaded DCs will migrate to lymph nodes to recruit and activate immune cells. Then activated B cells can promote tumor apoptosis through antibody-dependent cellular cytotoxicity and activated T cells can proliferate and differentiate into memory T cells or effector T cells. As a result, effector T cells will kill tumor cells directly or induce tumor cell apoptosis after traveling to TME. In 2010, the autologous DC-based prostate cancer vaccine Sipuleucel-T became the first human therapeutic cancer vaccine that was approved by the United States Food and Drug Administration (FDA) (Cheever and Higano, 2011).

#### 3.1.3 Cytokine therapies

Cytokines are small, soluble signaling proteins with a short half-life, which initiate the immune response to external stimuli directly and rapidly (Conlon et al., 2019). They are important for the immune cells to kill tumor cells owing to their participation in nearly all cellular responses such as regulation of immune cell proliferation and differentiation. Interferon (IFN)-α was the first approved cytokine drug for tumor immunotherapy by the FDA in 1986 (Berraondo et al., 2019). Despite its advantages, cytokine therapy causes a variety of side effects and has a narrow therapeutic window, which limits its clinical use. Thus, cytokines-based immunotherapy regulates a complex network of signals with multipotent, multisource, multiterminal, and multimodal activity.

#### 3.1.4 Adoptive cell transfer

Adoptive cell transfer (ACT) refers to the processing and modification of body’s immune cells with the use of genetic engineering (Rosenberg et al., 2008). Herein, the immune cells are cultured and amplified *in vitro* to enhance their tumor-specific killing function. As a result, they are reinjected into the patient’s body and ultimately kill the tumor cells. Compared with traditional surgical treatment, chemotherapy, or radiotherapy, immune cell therapy technology has remarkable advantages with regards to its curative effect, toxic and side effects, and tolerability. Clinically, adoptive immunotherapy includes tumor-infiltrating lymphocytes (TILs), T cell receptor (TCR) therapy, chimeric antigen receptor NK cell (CAR-NK) immunotherapy, and chimeric antigen receptor T cell (CAR-T) immunotherapy (Rohaan et al., 2019).

#### 3.1.5 Oncolytic virus therapies

Oncolytic viruses (OVs) are viruses that can effectively infect and destroy cancer cells. It involves the generation of special oncolytic viruses through genetic modification of some weakly pathogenic viruses that exist in nature, and then use the inactivation or defects of the tumor suppressor genes in the target cells to selectively infect the tumor cells. Finally, the oncolytic viruses replicate to generate large numbers of viruses and eventually destroy the tumor cells (Chiocca and Rabkin, 2014). Furthermore, it can continue to stimulate an immune response and attract more immune cells for the sustained killing of the remnant cancer cells. Due to the characteristics of the oncolytic virus (Pol et al., 2016), this kind of immune therapy can be administered systemically or locally to treat primary and metastatic tumors. In 2015, the FDA approved T-VEC as the first oncolytic herpes virus (a modified herpes simplex virus) for the treatment of melanoma, and it is currently one of the most successful oncolytic viruses. Ribas et al. (2017) found that the combined use of the oncolytic virus drug (T-VEC) and the anti-PD-1 antibody (Keytruda) for melanoma treatment showed a tumor remission rate as high as 62%, of which 33% were complete remissions. Previous data showed that the remission rate of Keytruda single-drug therapy was 47%, and the complete remission rate was 14%. These data indicate that the combined administration of T-VEC and Keytruda can further enhance the potency of Keytruda.

### 3.2 Synergistic antitumor immunotherapy

Since several cancer patients show low response to single immunotherapy treatments, and some of the cancers have low immunogenicity along with tumor immunosuppressive microenvironment, the immunotherapies may not be efficacious against such tumors (Lv et al., 2022). Therefore, combination therapy is an alternative approach to overcome these problems. In addition to dual synergistic immunotherapy, an increasing number of studies are exploring the combination of immunotherapy with chemotherapy, radiotherapy, phototherapy, and targeted therapy in the clinic, which may achieve better antitumor efficacy than single immunotherapy administration (Perez-Gracia et al., 2009; Hagan et al., 2020; Liu et al., 2021; Zhao et al., 2021). Simultaneously, immunotherapy may also make up for the shortcomings of other antitumor treatment strategies, which may prevent the development of therapeutic resistance (Tan et al., 2020).

#### 3.2.1 Synergistic immune checkpoint inhibition therapy

Unlike traditional and chemotherapy directly kill tumor cells, tumor immunotherapy aims to activate the body’s immune system and relies on the autoimmune function to kill tumor cells. Among them, immune checkpoint inhibitors play an important role during the treatment process of tumor evasion and immune surveillance. However, the clinical tumor response to its separate treatment is still only less than 40%, which greatly limits its clinical application. Thus, the combined application of ICI may show a better tumor treatment effect by increasing tumor immunogenicity and response rate of ICI themselves. PD-1 inhibits T cell activation by binding to its ligand PD-L1 or PD-L2. When CTLA-4 binds to CD80 and CD86 on the surface of antigen-presenting cells, it inactivates the T cells. Competitive inhibition of PD-1 or CTLA-4 with antibodies blocks the above-mentioned mechanism, thereby enhancing the killing activity of T cells. The CTLA-4 pathway mainly acts on T cells interaction with APCs, which majorly affects the activation of T cells and the function of the effector cells. The PD-1 pathway mainly acts on the tumor cells and activated lymphocytes and reduces the extent of T cell activation and cytotoxicity. Both the above pathways can influence each other but are relatively independent (Buchbinder and Desai, 2016). Moreover, combination therapy of PD-1 with CTLA-4 inhibitors targeting the above pathways can make it feasible to reverse the cold/hot tumors. The immune escape mechanisms of hot tumors usually include the up-regulation of immune checkpoint molecules (e.g., PD-L1) recruitment of regulatory T cells (Treg), and loss of surface antigen expression, etc, but they still have a large number of tumor-infiltrating lymphocytes. As a result, this type of tumor often responds well to PD-1 inhibitors. On the contrary, cold tumors lack lymphocyte infiltration, PD-L1 expression, and the host innate immune recognition process during the immune escape, so they neither recruit effector T cells nor respond to PD-1 inhibitors. However, it was reported that when CTLA-4 inhibitors were applied to such cold tumors, CD3^+^/CD4+ and CD3^+^/CD8+ T cells could be recruited and even fully activated to increase the infiltration of lymphocytes in the tumor microenvironment and upregulate INF-γ, thereby up-regulating the expression of PD-L1 to resensitize the tumor cells to PD-1 inhibitors (Wei et al., 2017; Wu et al., 2019; Rupp et al., 2022). Therefore, the combined dual administration of CTLA-4 and PD-1 inhibitors may have a synergistic effect, or be effective in patients with negative expression of one ligand/receptor. Other combination therapies like LAG-3 and PD-L1 inhibitors are still under clinical trials, and the findings from these trials may further broaden the application of ICI in cancer treatment (Robert, 2021).

#### 3.2.2 Synergistic combination of chemotherapy and cancer immunotherapy

**FIGURE 4:**
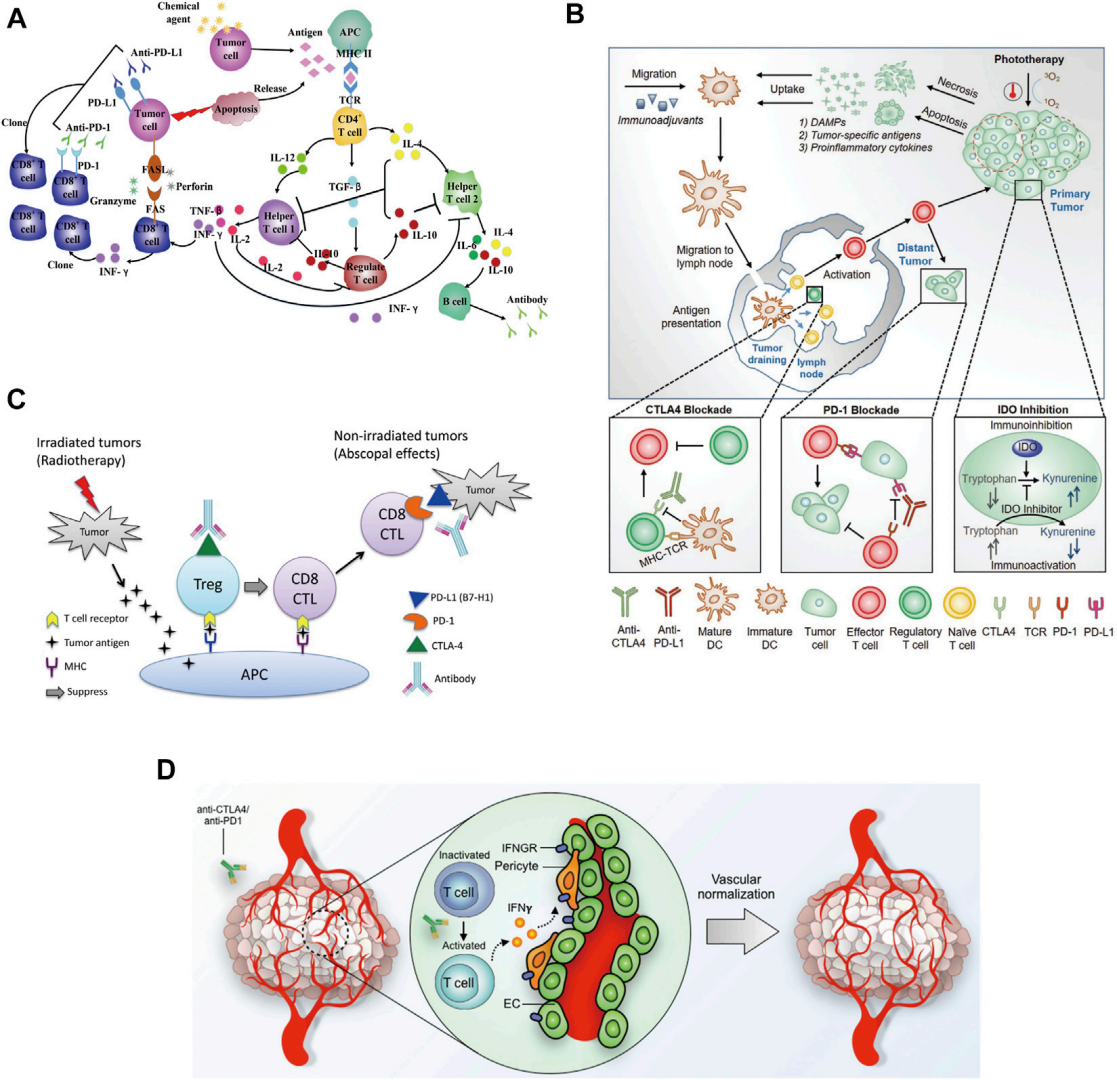
**(A)** Chemotherapeutic agents influence cytokines network in the antitumor immune system (Luo and Fu, 2016). **(B)** An overview of the combination of phototherapy and immunotherapy (Ng et al., 2018). **(C)** Potential mechanism of action of the combination of radiotherapy and immunotherapy (Mansfield et al., 2015). **(D)** Potential mechanism of immunotherapy-mediated tumor vascular normalization (Liu et al., 2019).

The anti-tumor effects of chemotherapy combined with immunotherapy are multifaceted [Figure 4A, (Luo and Fu, 2016)]. Chemotherapy augments the effect of immunotherapy by enhancing tumor cell immunogenicity, suppressing immunosuppression, and inducing an antitumor immune response (e.g., immunogenic cell death, ICD). In addition, immunotherapy reverses the chemotherapy resistance of tumor cells, which improves the chemosensitivity of tumor cells and reduces their toxicity. In May 2017, the United States FDA accelerated the approval of the anti-PD-1 antibody pembrolizumab, pemetrexed and carboplatin for the first-line treatment of advanced or metastatic non-small cell lung cancer (NSCLC) (Qu et al., 2020). The results of the clinical trials showed that the group that received the combination of chemotherapy and immunotherapy had a significantly better objective remission rate (55% vs. 29%) and prolonged progression-free survival (PFS) (13.0 vs. 8.9 months) than the group that received the chemotherapy alone. At the 2021 American Society of Clinical Oncology (ASCO) annual meeting, the KEYNOTE-590 study that was presented was the first one reporting the international first-line application of anti-PD-1 antibody combined with cisplatin and 5-fluorouracil for the treatment of esophageal cancer. Moreover, the published results from a subgroup analysis in China were completely consistent with the results of the global study. Regardless of the expression of PD-L1, immunotherapy combined with chemotherapy may bring survival benefits for unresectable locally advanced or metastatic esophageal cancer patients receiving the first-line treatment (Kato et al., 2019; Li et al., 2021a). This result had a very significant and far-reaching impact on the treatment of esophageal cancer. At present, there are still other treatment strategies combining chemotherapy and immunotherapy in the clinic, However, further clinical evidence is needed to understand if those strategies can produce similar therapeutic effects or can be used for the treatment of multiple cancer types.

#### 3.2.3 Synergistic combination of phototherapy and cancer immunotherapy

Phototherapy is a promising alternative approach that offers an elegant solution to eradicate tumors through the simple application of light irradiation (Wang et al., 2021). Meanwhile, it is also associated with antitumor immune response by inducing immunogenic cell death and enhancing the antitumor immunity. Phototherapy is comprised of photothermal and photodynamic therapy. In general, photothermal therapy (PTT) leads to cell death by necrosis (Hou et al., 2020), while photodynamic therapy (PDT) typically induces cellular apoptosis. Herein, the combination of phototherapy with cancer immunotherapy has been demonstrated to promote synergistic outcomes, promote cancer regression, and even induce immunologic memory [Figure 4B, (Ng et al., 2018)]. With promising discoveries in combination therapeutic approaches, it has been possible to achieve higher levels of proinflammatory cytokines, improved migration of dendritic cells, and an increased ratio of tumor-infiltrating cytotoxic T cells against regulatory T cells, making their clinical applications lucrative. Chen et al. reported that photothermal therapy could promote tumor infiltration of CAR-T cells and potentiate its antitumor activity (Chen et al., 2019). Desmoplastic structures and immunosuppressive microenvironment usually accounted for the reduced efficacy of CAR-T cells in solid tumors. Mild hyperthermia of the tumors reduced its compact structure and interstitial fluid pressure, increased blood perfusion, released antigens, and promoted the recruitment of endogenous immune cells. Hence, the combination of mild photothermal therapy with the adoptive transfer of CAR-T cells could potentially increase the therapeutic index of these cells in solid tumors. It was reported that infusing chondroitin sulfate proteoglycan-4 (CSPG4)-specific CAR-T cells into NOD SCID gamma mice engrafted with the human melanoma WM115 cells showed superior antitumor activity following photothermal ablation of the tumor. These findings suggested that photothermal therapy facilitated the accumulation and effector function of CAR-T cells within solid tumors. But future advances involving the combination of phototherapies with other strategies remain to be explored, which may help overcome the immunosuppressive environment at the tumor site (Li et al., 2021b; Huang et al., 2021c).

#### 3.2.4 Synergistic combination of radiotherapy and cancer immunotherapy

The radiotherapy mechanism uses high-energy particles or waves such as X-rays to destroy or damage the cancer cells (Baskar et al., 2012). Radiotherapy causes radiation damage to the tumor and its surrounding normal tissues and cells (Barnett et al., 2009). Both normal and tumor cells can repair this damage, but normal cells are more capable of repairing radiation damage than tumors and these damages prevent cancer cells from growing and proliferating and ultimately killing them. Unlike chemotherapy, which exposes the whole body to anticancer agents, radiotherapy is usually localized to the tumor site. In most cases, it only targets and affects one part of the body, which is the treated area (Formenti and Demaria, 2009). In the PACIFIC study, durvalumab (10 mg/kg) was used for consolidation therapy in addition to radiotherapy and chemotherapy for patients with stage III NSCLC (non-small cell lung cancer) (Antonia et al., 2017). Compared with the placebo group, the median progression-free survival (mPFS) was significantly prolonged (16.8 vs. 5.6 months). But, so far, most of the studies reporting on radiotherapy combined with immune checkpoint inhibition are still retrospective studies or small group studies. A better strategy for the combination of radiotherapy and immunotherapy needs to be developed and verified by further clinical trials [Figure 4C, (Mansfield et al., 2015)].

#### 3.2.5 Synergistic combination of vascular targeted therapy and cancer immunotherapy

Anti-angiogenesis therapy is one of the standard treatments for a variety of solid tumors (such as non-small cell lung cancer, breast cancer, colorectal cancer, etc.) in the clinic. Compared with general molecular targeting strategies, it can only be selectively administered based on specific biomarkers (Al-Abd et al., 2017). It is used for specific tumor classification, which is suitable for a wider group of patients. For liver cancer treatment, there is no specific single target that could serve as a biomarker and treatment with vascular targeted therapy could induce the production of many drug-resistant enzymes like phosphorylated extracellular signal-regulated kinase in these patients (Llovet et al., 2021). Therefore, multi-targeted drugs are needed for the treatment of liver cancer. Clinically approved drugs such as sorafenib which directly target the VEGF signaling pathway, act on multiple targets at the same time and show an anti-angiogenic effect through multiple mechanisms. Unfortunately, the actual effect of this treatment is not always satisfactory (Wilhelm et al., 2008). On the other hand, although the survival of liver cancer patients treated with PD-1 inhibitors as a single agent showed was prolonged, no statistically significant results were observed. Since single drugs tend to have a beneficial effect, there may be synergistic therapeutic combination strategies. Furthermore, normalizing the tumor vasculature has been shown to improve the efficacy of cancer immunotherapies, and emerging studies also suggest that enhanced immune stimulation, in turn, improves tumor vascular normalization, forming a mutually supportive loop [Figure 4D, (Liu et al., 2019)]. In the clinical study named IMbrave150, a total of 501 patients were enrolled globally. The patients received Atezolizumab (PD-L1 monoclonal antibody) and Bevacizumab (targeted for VEGF), simultaneously. The results showed that the median progression-free survival of the combined group was 6.8 months, and that of the sorafenib group was 4.3 months, reducing the risk of progression by 41%. Therefore, the combination of vascular targeted therapy with immunotherapy may bring revolutionary benefits to liver cancer patients (Finn et al., 2020). At the same time, related studies have shown that Vemurafenib (BRAF inhibitor) combined with Cobimetinib (MEK1/2 inhibitor) and Atezolizumab could significantly prolong the progression-free survival (PFS) of melanoma patients (Subbiah et al., 2020). Thus, the immune mediated vascular normalization opens up the possibility for identifying new cancer treatment strategies combining vascular targeting agents and immunotherapies.

## 4 Nano-drug delivery systems for synergistic antitumor immunotherapies

Clinically, synergistic antitumor immunotherapy is gaining wide attention nowadays. However, there are still many difficulties and challenges in controlling the proportion of the combined drugs, synergistic onset time, and adverse reactions (Senapati et al., 2018). Therefore, the rational design of the nano-delivery system to construct a synergistic combination treatment strategy is also conducive to promoting the development of personalized treatment and precision medicine. The co-delivery of immunotherapeutic drugs and other therapeutic drugs such as photosensitizers, chemotherapeutics and immune adjuvants have plenty of advantages, such as controlling the proportion of the combined drugs, prolonging the blood circulation time of drugs, realizing the targeted delivery of drugs at the tumor site and improving the tumor microenvironment (Huang et al., 2021d; Yang et al., 2021). But, for different combination regimens, specific nano-drug delivery systems are often required to meet the requirements of delivery and improve therapeutic efficacy (Patra et al., 2018)

### 4.1 Nano-drug delivery systems for combination of multiple immunotherapy strategies

**FIGURE 5:**
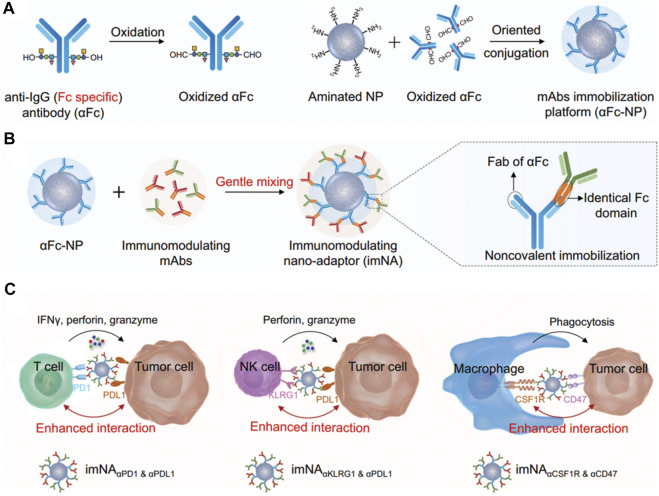
**(A–C)** Schematic illustrating the design of imNAs and their potential to improve antibody-based cancer immunotherapy (Jiang et al., 2021).

In recent years, tumor immunotherapy, especially immune checkpoint blocking therapy, has rapidly advanced and is profoundly changing the treatment strategy for malignant tumors. Monoclonal antibodies have been widely used in tumor immunotherapy, but they suffer from drawbacks such as immune escape and drug resistance, which influence their anti-tumor efficacy in the clinic (Weiner et al., 2010). When compared with monoclonal antibodies, bispecific antibodies (BsAb) play a unique role in mediating the killing of tumor cells by the immune cells through their dual target signal blocking mechanism, which effectively prevents tumor cell drug resistance (Brinkmann and Kontermann, 2017). At the same time, the BsAb has stronger specificity, targeting ability and reduced off-target toxicity. However, ensuring the stability of BsAb antibodies, balancing the expression of the two antibodies and exploring an optimal format of the hinge region remain the major challenges for the development of novel therapeutic BsAbs. Therefore, the nanoplatform technology serves as an important method for the development of bispecific antibodies. Because the Fc segment of the therapeutic monoclonal antibody drugs in preclinical trials is highly consistent, Jiang et al. (2021) innovatively proposed the use of antibodies [anti-IgG (Fc specific) antibody, αFc] that can specifically recognize the Fc segment of antibodies to construct a universal antibody immobilization platform [Figures 5A–C, (Jiang et al., 2021),], through simple physical mixing of a new type of bispecific nanobody named “immunomodulating nano-adaptor.” This nano-adaptor could be prepared conveniently, efficiently and controllably to realize the multivalency, bispecificity, and multifunctionality of the monoclonal antibody. Compared with conventional monoclonal antibody drugs, the nano-adaptor not only regulates the function of immune cells but also significantly enhances the interaction of immune cells (including T cells, macrophages, natural killer cells, etc.) with the tumor cells, effectively increasing the antitumor effects of the cloned antibody drugs. Nano-drug delivery system is an important technique that combines multiple immunotherapies and prevents the tumor immune escape arising from the single immune treatment. Furthermore, the co-delivery of multiple antibodies may induce further synergistic effects, similar to bi-specific antibodies and multi-specific antibodies, which is a highly promising strategy (Parker et al., 2019; Bai et al., 2020).

Nano-drug delivery systems for the combination of chemotherapy and cancer immunotherapy.

**FIGURE 6:**
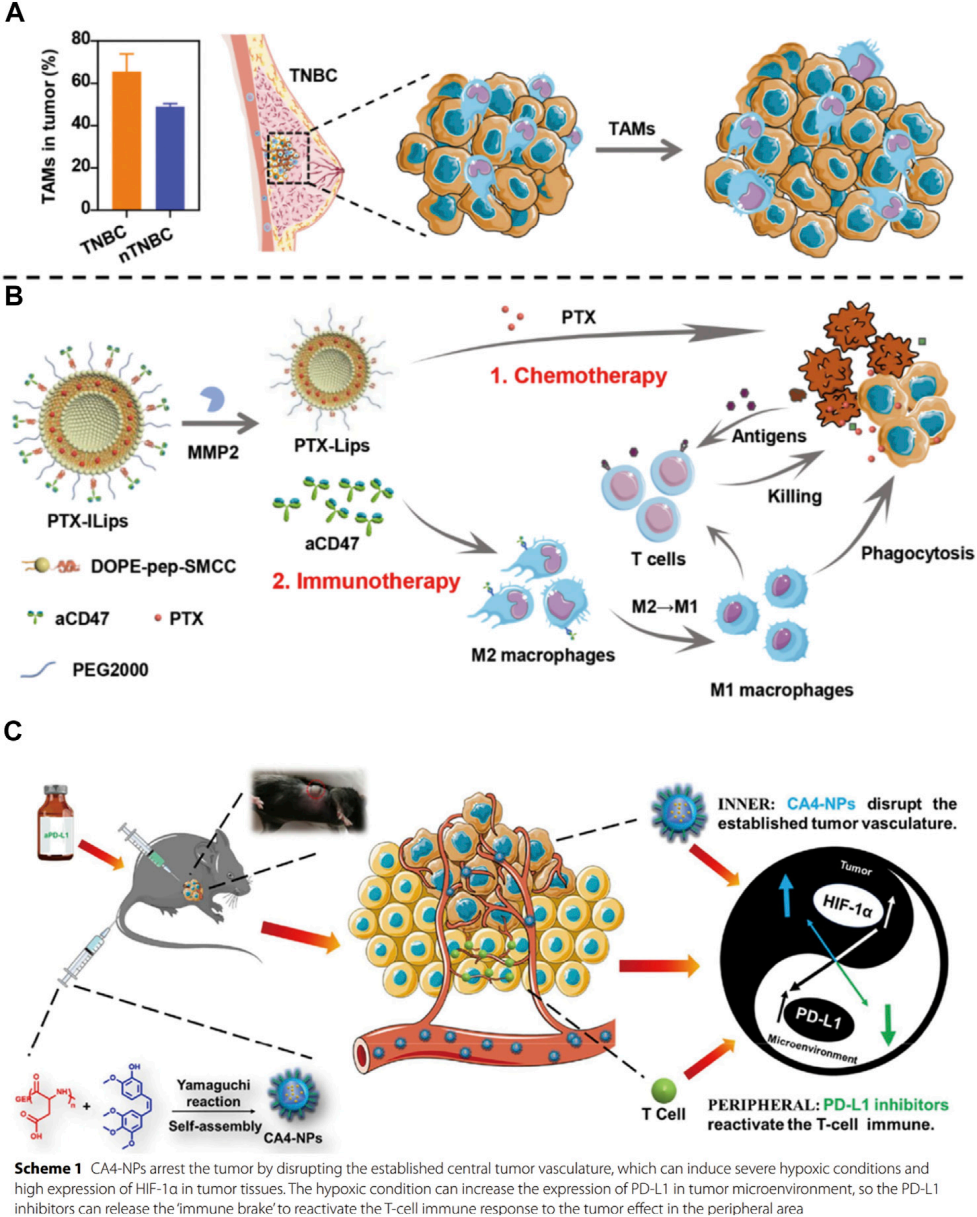
**(A)** Tumor-associated macrophages (TAMs) promote tumor growth in triple-negative breast cancer (TNBC) and **(B)** PTX-ILips designed for the enhanced efficacy of immunochemotherapy against TNBC (Chen et al., 2021). **(C)** Mechanism of action of the combination of CA4-NPs and aPD-L1 (Zhao et al., 2021).

The strategy of combining immunotherapy with chemotherapy is a classic approach for antitumor therapy. The nano drug delivery system used for its co-delivery needs to be able to load the antibody drugs along with the small molecule chemotherapeutic drugs to achieve the controlled release of both the drugs at the tumor site and improve both the pharmacokinetics and biodistribution of these drugs (Din et al., 2017; Senapati et al., 2018). These carriers include liposomes (Zununi Vahed et al., 2017), polymer micelles (Krishnamurthy et al., 2015), inorganic silica nanoparticles (Chen et al., 2014), metal-organic framework materials and so on (Ding et al., 2020). Triple-negative breast cancer (TNBC) is an aggressive malignancy with a high recurrence rate and poor outcomes in the clinic. Because tumor-associated macrophages (TAMs) were found to be enriched in TNBC, Chen *et al.* (2021) designed and synthesized a matrix metalloprotease 2 (MMP2) responsive integrated strategy [Figures 6A,B, (Chen et al., 2021). It could deliver paclitaxel (PTX) and anti-CD47 (aCD47) using detachable immune liposomes (ILips). In the TNBC microenvironment, the “two-in-one” ILips facilitated the MMP2-responsive release of aCD47 to efficiently polarize the M2 macrophages toward the M1 phenotype to enhance the phagocytosis of tumor cells and activate the systemic T-cell immune response. Together with the immune effect, the detached PTX-loaded liposomes were internalized by the MDA-MB-231 cells to synergistically inhibit tumor cell proliferation and metastasis. In the TNBC-bearing mouse model, PTX-loaded ILips demonstrated superior antitumor efficacy and inhibited tumor recurrence. This integrated strategy represented a promising approach to synchronously enhance the immune response and tumor-killing effects, improving the therapeutic efficacy against TNBC. In addition, researchers also tried to achieve immunochemotherapeutic therapy by designing different ways to administer chemical drugs and immunotherapy through nano-drug delivery systems. Zhao et al. (2021) used poly (L-Aspartic acid)-*b*-poly (ethylene glycol) loaded with combretastatin A4 through ester bond. It could be self-assembled to form nanomicelles (CA4-NPs), which were aimed to significantly disrupt new blood vessel formation in the tumor tissues for targeted liver cancer therapy [Figure 6C, (Zhao et al., 2021)]. Here, CA4-NPs were mainly distributed at the tumor site because of the triple targeting effects, namely EPR effect, acid-sensitive (pH = 5.5) effect in the tumor microenvironment, and good selectivity of CA4 for the central tumor blood vessel. Considering that CA4-NPs might induce severe hypoxic conditions resulting in the high expression of HIF-1α by the tumor tissues, which could induce the overexpression of PD-L1, the researchers also used an anti-PD-L1 antibody (aPD-L1) to prevent immunosuppression. This way of complementary combination was able to achieve an ideal therapeutic effect at tumor sites where CA4-NPs and aPD-L1 could respond to the inner area and peripheral area, respectively. As a result, a significant decrease in tumor volume and weight were observed in the combination group that received CA4-NPs and aPD-L1 compared with CA4-NPs or aPD-L1 monotherapy in subcutaneous Hepa1-6 hepatic tumor models. Overall, in recent years, benefiting from the results of a large number of clinical trials of immunotherapy combined with chemotherapy, this kind of strategy has become the first-line treatment regimen for many cancers, including non-small cell lung cancer, small cell lung cancer, head and neck cancer, breast cancer, etc (Salas-Benito et al., 2021). The application of nano-delivery systems in combination treatment strategies can further improve the effect of antitumor therapies, and enable the development of further therapies combining immunotherapy and chemotherapy.

### 4.2 Nano-drug delivery systems for combined photothermal therapy and cancer immunotherapy

**FIGURE 7:**
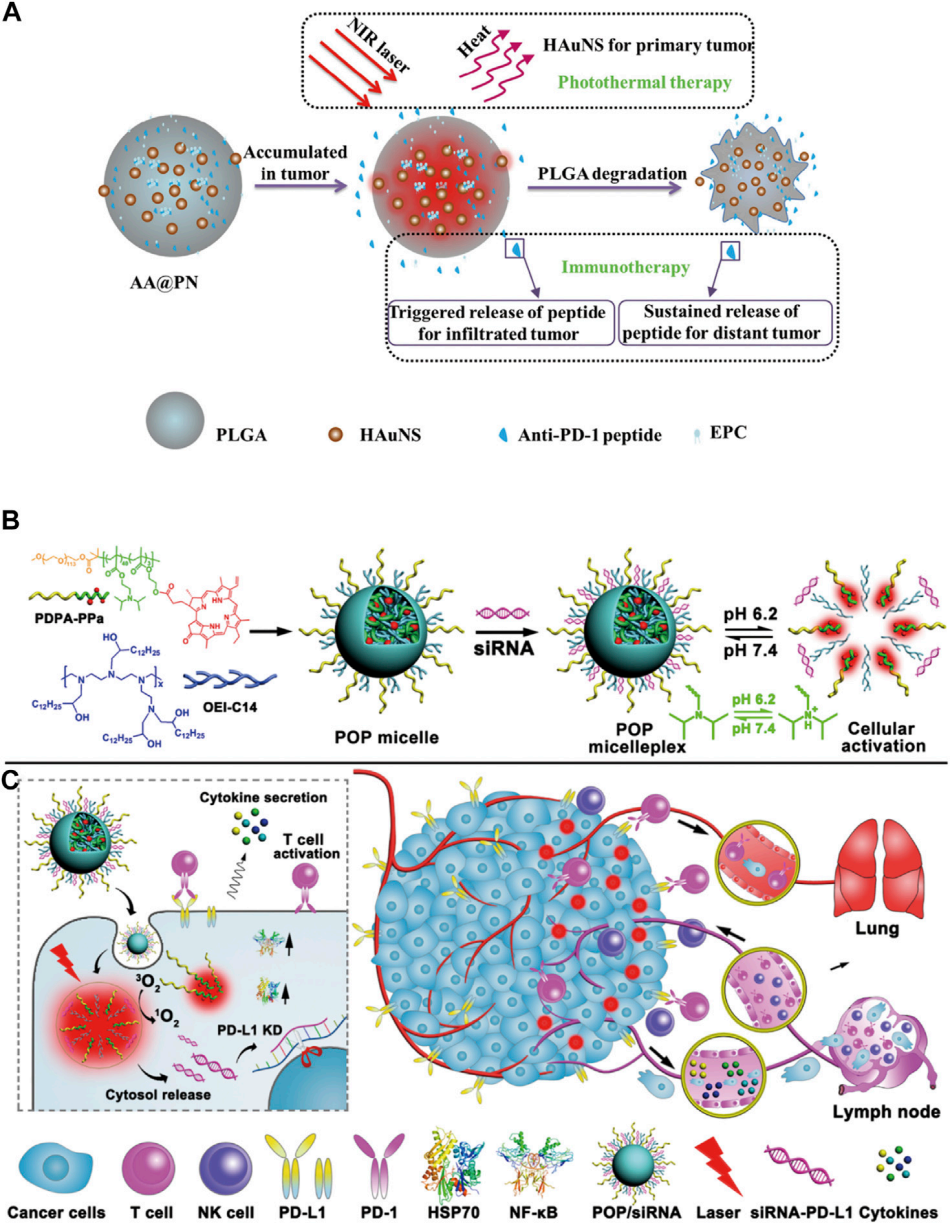
**(A)** Scheme showing the preparation and structure of AA@PN as well as its combined therapeutic modalities (Luo et al., 2018). **(B,C)** Cartoon schematic of POP−PD-L1 micelleplex mediated photodynamic cancer immunotherapy (Wang et al., 2016).

Nano drug delivery systems used for combined immunotherapy and photothermal therapy mostly contain nanomaterials with near-infrared photothermal conversion function, including inorganic materials such as gold (Liu et al., 2018), copper sulfide (Guo et al., 2014), organic materials such as heptamethine (ICG, IR780, IR820) (Alves et al., 2022), and some carbon quantum dots (Zhang et al., 2018b). A new strategy was proposed by Luo *et al.* (2018) that combined PD-1 blockers with photothermal ablation for treating malignant tumors by co-encapsulating anti-PD-1 peptide and hollow gold nanoshell into biodegradable PLGA [Figure 7A, (Luo et al., 2018),]. The results showed that the slow and continuous release of anti-PD-L1 antibodies from PLGA could be achieved from 0 to 40 days, and this release was easily accelerated by illumination with a near-infrared (NIR) laser. Overall, a clear killing effect on distant tumor cells was observed after the combination therapy, reflecting the activation of the antitumor immune response. The use of nano-systems to deliver photothermal materials was conducive to its improved long-term photothermal effects at the tumor site. Meanwhile, continuous immune responses were triggered by immunogenic cell death to enhance the antitumor immunity, which had a synergistic therapeutic effect with immunotherapy.

In the second kind of phototherapy, photodynamic therapy, the excited photosensitizer releases reactive oxygen species in the tumor tissues to induce tumor cell apoptosis and it is also associated with anti-tumor immune response. However, the immunosuppressive tumor microenvironment limits the immune response induced by PDT. Thus, it is necessary to combine PDT with immune checkpoint inhibitors and immunoadjuvant therapy for synergistic treatment of the tumors. Moreover, PDT also requires photosensitizers to accumulate at the tumor site, therefore, the nano delivery systems are required to deliver them. Some nano-delivery materials are inherently photosensitive. Therefore, the photosensitizer can be loaded into the nano drug delivery system, or the carrier material with photosensitivity can be used to prepare the nanoparticles. At present, the photosensitizers commonly used for combination with immunotherapy include ICG (Shirata et al., 2017), Ce6 (Li et al., 2014), temoporfin (Tan et al., 2010), pheophorbide A (PPa) (Tang et al., 2010), etc. In the study from Wang *et al.* (2016), they demonstrated that PDT-mediated cancer immunotherapy could be augmented by PD-L1 knockdown in the tumor cells [Figures 7B,C, (Wang et al., 2016)]. Hence, they designed a versatile micelleplex by integrating an acid-activatable cationic micelle, PPa, and small interfering RNA (siRNA). The micelleplex was inert at physiological pH conditions and was activated only upon internalization into the acidic endocytic vesicles of the tumor cells for fluorescence imaging and PDT. These results showed that compared to PDT alone, the combination of PDT and siRNA showed significantly enhanced efficacy for inhibiting tumor growth and distant metastasis in a B16-F10 melanoma xenograft tumor model. It has been suggested that acid-activatable micelleplexes utilizing PDT-induced cancer immunotherapy were more effective when combined with siRNA-mediated PD-L1 blockade, which could provide a general strategy for enhancing the therapeutic efficacy of PDT.

Nano-drug delivery systems for combined radiotherapy and cancer immunotherapy.

**FIGURE 8:**
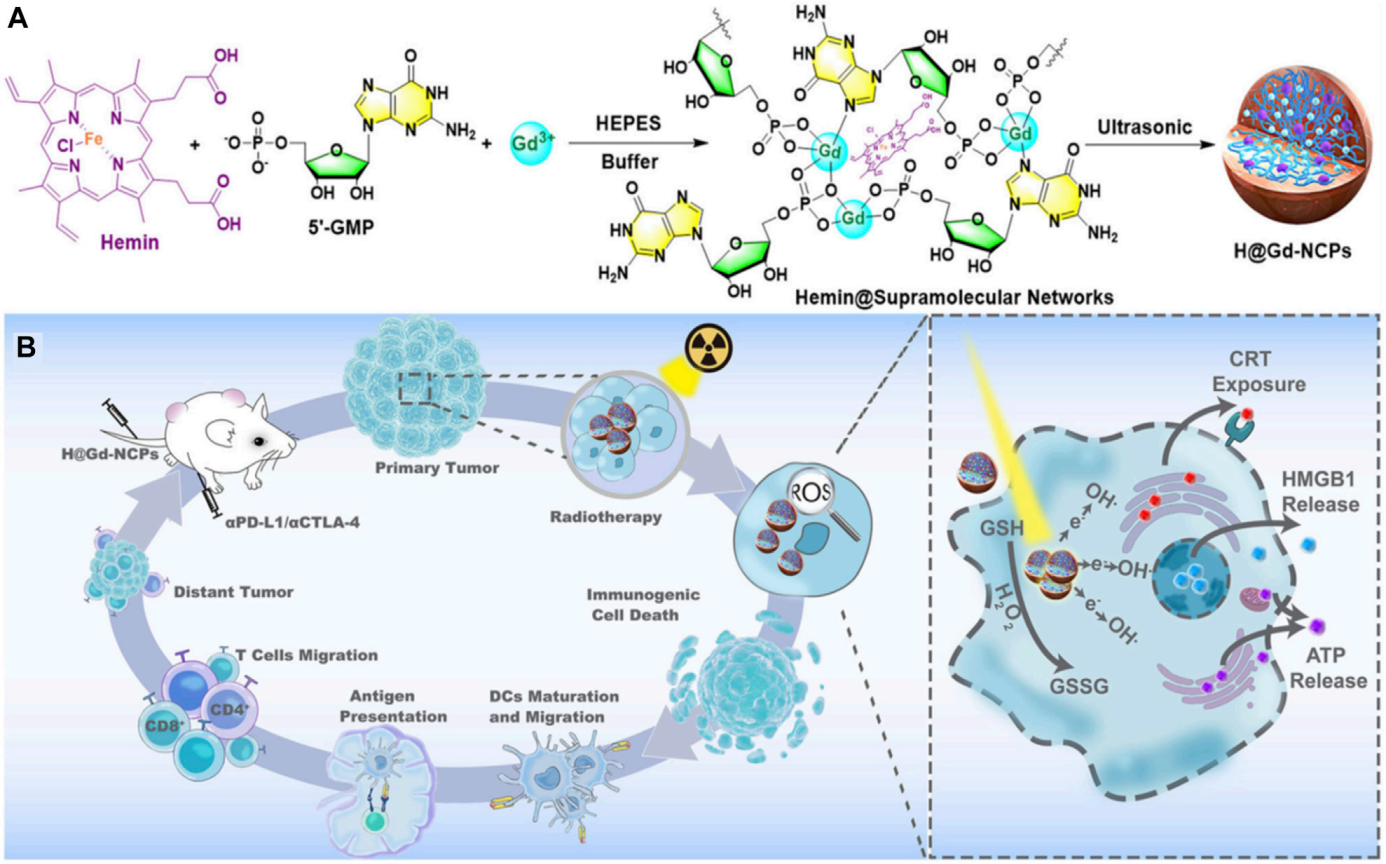
**(A,B)** The preparation and mechanism of action of H@Gd-NCPs (Huang et al., 2021c).

Radiotherapy is currently one of the most widely used cancer treatment methods in the clinic, but poor specificity of traditional *in vivo* radiotherapy, short-term effects in the tumor tissue and side effects on normal tissues, limit its wide application. Some multifunctional nanomaterials themselves can be used as radiotherapy sensitizing agents and radiation dose enhancers to effectively improve the efficacy of radiotherapy against the tumor lesions, thereby overcoming the dose tolerance constraints of healthy tissues and enhancing radiosensitization (Yu et al., 2022). On the other hand, nanomaterials can be used as drug carriers to load different kinds of drugs to help regulate signaling pathways and cell cycles, inhibit DNA repair mechanisms, indirectly promote tumor cell apoptosis or selectively kill tumor cells and modulate the tumor microenvironment, which could improve the overall radiotherapy sensitivity (Kwatra et al., 2013). Huang et al. (2021a) constructed nano-scale gadolinium-based coordination polymers (H@Gd-NCPs) by combining gadolinium (Gd3+), 5′-guanylic acid (5′-GMP) and one peroxidase activity agent-Hemin [Figures 8A,B, (Huang et al., 2021b)]. Due to the existence of “Gadolinium,” it could not only be used as an excellent nuclear magnetic contrast agent, but also as a high-order atom Gd to effectively deposit X-rays at a relatively low dose of radiotherapy, and increase the effect of local radiotherapy. Moreover, Hemin encapsulated within H@Gd-NCPs could enhance the peroxidase-like properties to utilize the overexpressed H_2_O_2_ in the tumor microenvironment, leading to GSH depletion. The integration of ROS enhancement and GSH depletion eventually amplifies irradiation-mediated oxidative stress and induces ICD.

Nano-drug delivery systems for the combination of targeted therapy with cancer immunotherapy.

**FIGURE 9:**
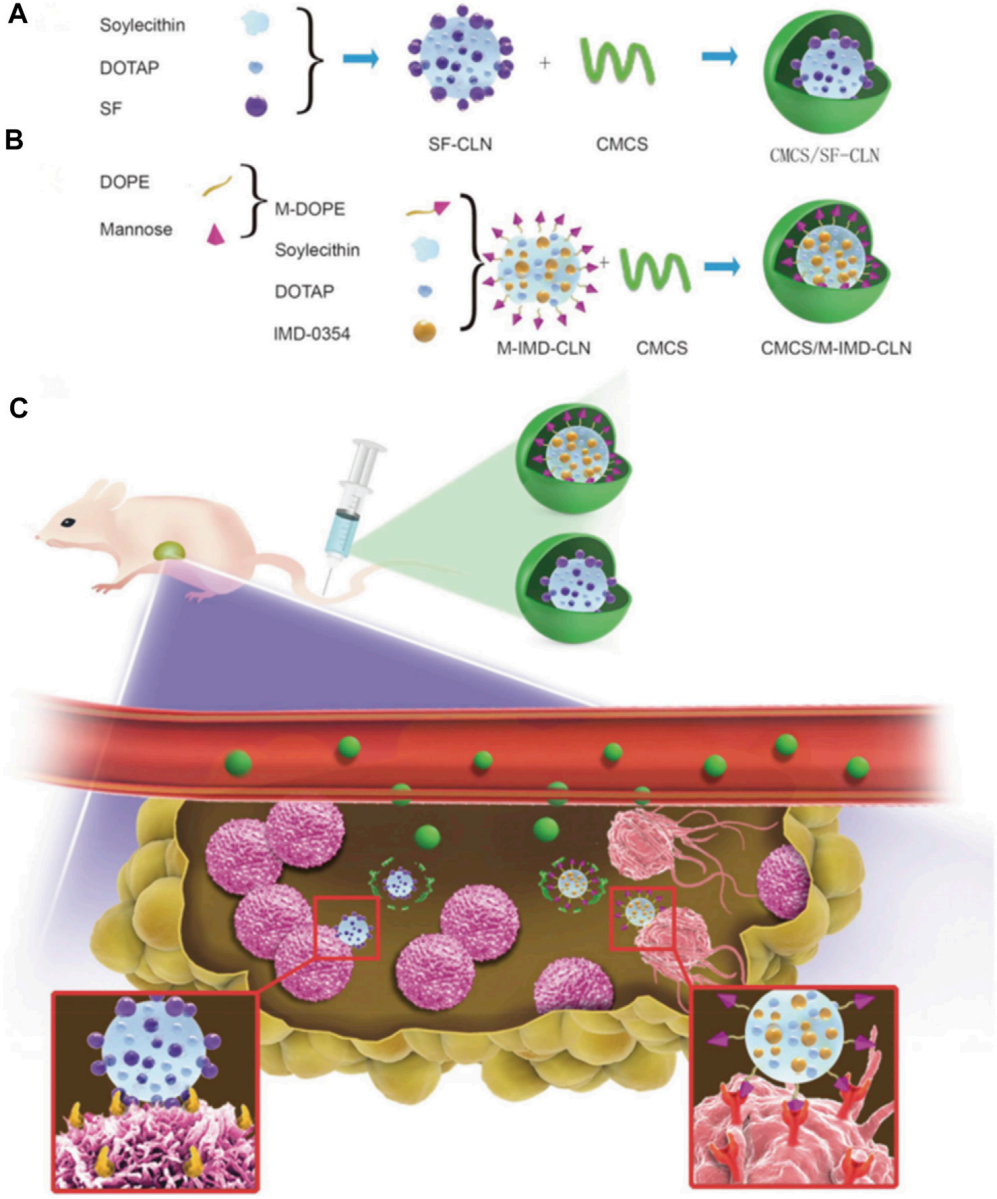
**(A–C)** Scheme of twin-like core-shell nanoparticles (CMCS/SF-CLN + CMCS/M-IMD-CLN) for synchronous biodistribution and targeted delivery to enhance chemo-immunotherapy (Wang et al., 2019).

Based on the constantly changing anti-angiogenesis mechanisms and related targets, researchers have shifted from solely focusing on vascular endothelial cells and other components of the blood vessel itself to the components of tumor extracellular matrix (such as collagen, hyaluronic acid), tumor-related fibroblasts cells, immune cells and stem cells in targeted tumor treatment area (Richards et al., 2010; Henke et al., 2019; Baghban et al., 2020). In addition, it is also possible to explore further combination treatment strategies of angiogenesis targeted therapy with other therapeutic methods such as immunotherapy, for clinical application. During the process of developing combined targeted therapies, researchers found that nano-delivery systems can exert their unique synergistic effects. In addition to the characteristics of long circulation and good tumor site permeability to improve the delivery efficiency, they can also integrate various functional components to actively target the tumor blood vessels, improve hypoxia within the TME, and regulate immunity, resulting in a “cocktail effect” (Abou Khouzam et al., 2021; Janji and Chouaib, 2022). Tumor-associated macrophage (TAM)-based immunotherapy has been presented as a promising strategy for cancer therapy (Kumari and Choi, 2022). Therefore, the combination of TAM-based immunotherapy with sorafenib (SF), one of the most important multikinase targeted inhibitors, may be more effective for treating hepatocellular carcinoma (HCC). In a previous study from Wang *et al.* (2019), the researchers designed and synthesized twin-like core-shell nanoparticles (TCN) for synchronous biodistribution and separated cell-targeted delivery of SF and TAM re-polarization agents IMD-0354, to cancer cells to enhance tumor-localized chemo-immunotherapy [Figure 9, (Wang et al., 2019)]. The analysis of the antitumor efficiency *in vivo* and phenotypic analysis of the TAMs in the tumor tissues showed that the combination therapy group exhibited superior synergistic antitumor efficacy and M2-type TAM polarization ability as compared with the SF mono-therapy group in Hepa1-6 tumor-bearing mice. Consequently, TCN enabled the co-administration of the drug combination and served as a nano-drug delivery system, and showed great potential for application in tumor targeted-immunotherapy in the clinic.

## 5 Conclusion

To conclude, although cancer immunotherapy has garnered wide attention clinically, it still suffers from several limitations such as generating immune tolerance and escape, immune side effects and poor tumor targeting, etc. The application of nano-drug delivery systems may significantly alleviate the above problems by enhancing the stability and lengthening the circulation time, which is beneficial for cellular uptake to stimulate the antitumor immune response. An increasing number of studies have revealed that the combined treatment with nano-immunotherapy had more advantages, such as further improvement in the therapeutic efficacy, reduced side effects, reduced immune tolerance and escape. However, such treatment strategies also suffer from different limitations such as the toxic and side effects mediated by the nano-drug carrier itself during the process of treatment *in vivo*. Besides, it is reported that although a large amount of anti-tumor studies showed encouraging efficacies in animal models, the clinical and translational application of nano-drug delivery systems for synergistic tumor immunotherapy is still in progress and many issues still need to be addressed. We believe that further breakthroughs and discoveries will appear in the tumor immune synergistic therapy with nano-drug delivery systems, which may help expand their clinical applications in the future.
